# Research on Volleyball Video Intelligent Description Technology Combining the Long-Term and Short-Term Memory Network and Attention Mechanism

**DOI:** 10.1155/2021/7088837

**Published:** 2021-10-14

**Authors:** Yuhua Gao, Yong Mo, Heng Zhang, Ruiyin Huang, Zilong Chen

**Affiliations:** ^1^Guangzhou Sport University, Guangzhou, Guangdong 510500, China; ^2^Guangdong Baiyun University, Guangzhou, Guangdong 510450, China; ^3^Yingshan County No. 1 Middle School, Hubei, Yingshan 438700, China

## Abstract

With the development of computer technology, video description, which combines the key technologies in the field of natural language processing and computer vision, has attracted more and more researchers' attention. Among them, how to objectively and efficiently describe high-speed and detailed sports videos is the key to the development of the video description field. In view of the problems of sentence errors and loss of visual information in the generation of the video description text due to the lack of language learning information in the existing video description methods, a multihead model combining the long-term and short-term memory network and attention mechanism is proposed for the intelligent description of the volleyball video. Through the introduction of the attention mechanism, the model pays much attention to the significant areas in the video when generating sentences. Through the comparative experiment with different models, the results show that the model with the attention mechanism can effectively solve the loss of visual information. Compared with the LSTM and base model, the multihead model proposed in this paper, which combines the long-term and short-term memory network and attention mechanism, has higher scores in all evaluation indexes and significantly improved the quality of the intelligent text description of the volleyball video.

## 1. Introduction

With the continuous development of big data, computer computing power, and machine learning model, video description technology has set off a research upsurge again. Video description technology is an interdisciplinary research problem. It is an exploration of the expansion of deep learning technology to the field of multidata after making outstanding achievements in the fields of natural language processing, speech recognition, and computer vision [1]. It can be widely used in video retrieval, intelligent security, human-computer interaction, virtual reality, and helping the blind understand films and videos. It has high application value and practical significance. Among all kinds of multimedia data, video has become an important carrier of information dissemination in today's society because of its large amount of information and rich content [2]. With the rapid development of video sensors, we can easily collect a large number of complex video data, and how to use natural language to describe the stored information has become an urgent problem to be solved. The task of using natural language to describe a video is very simple for normal people, but it is a very difficult task for computers. It requires that the proposed method can span the semantic gap from low-level pixel features to high-level language. The existence of the semantic gap brings great difficulties for the computer to automatically describe the video. The existing video description is usually carried out by manually labeling video data. This method is inefficient and often subjective, and it is easy to ignore many details [3]. Therefore, it is of great practical significance to find an efficient and objective way to describe the video to help people retrieve the video more quickly and conveniently.

With the rapid development of deep learning, researchers began to apply this technology to video description. Current research studies generally use the convolutional neural network (CNN) structure as the encoder to extract visual information and the long short-term memory (LSTM) network structure as the decoder to predict the description sentence [4]. Although these methods avoid the subjectivity of manual annotation to a certain extent, due to the lack of depth of language learning information and less grammatical supervision when generating description sentences, the predicted description sentences will have sentence errors such as missing predicates and loss of visual information. At the same time, sports video occupies an important position in the field of video description because of its huge audience. In particular, volleyball videos often present high-speed and detailed characteristics, which increase the difficulty of understanding the intelligent description of visual targets of video sensors [5]. Therefore, a video sensor processing method combining the long-term and short-term memory network and attention mechanism is proposed for the intelligent description of the volleyball video. The introduction of the attention mechanism can make the model pay much attention to the significant areas in the image/video when generating sentences, quickly identify the target, and effectively solve the loss of visual information.

Aiming at the problems of the lack of visual information, syntax error, and strong subjectivity in video description methods in existing video sensors, this paper proposes a method combining the long-term and short-term memory network and attention mechanism to describe the volleyball video. In the first section, the research background and significance of video description are briefly described. The second section briefly describes the research status of the video description of video sensors, discusses the problems to be solved in the current video description methods, and makes a general introduction to the research work and research methods of this paper. The third section first introduces the long-term and short-term memory network and attention mechanism and then gives the application in volleyball video description combined with the long-term and short-term memory network and attention mechanism model. In the fourth section, the datasets for training and testing are selected, and the evaluation indexes of the model recognition effect are determined. Then, a series of control experiments are set up to test the effectiveness of the attention mechanism model combined with the long-term and short-term memory network in the field of video description. The fifth section briefly summarizes the main conclusions of the article.

## 2. The Related Works

Thanks to the research and development of sensor technology, embedded technology, machine translation, image description, and the expansion of annotated video datasets in recent years, the video description task in video sensors has also attracted extensive attention of researchers, and the research of video description methods has also made great progress [6].

Early video description methods mainly generated sentences based on predefined templates. The sentences describing the video were first divided into several parts, each part should be aligned with the visual content, and then the words detected from the vision were filled into the predefined templates. Kojima et al. selected the most appropriate verbs and objects by detecting the human posture; then, the content displayed by the action semantics is corresponding to the features extracted from the video image, and finally filled the detected syntactic components into common case templates [7]. Rohrbach et al. first generated a rich semantic representation of visual content. They simulated the relationship between different components of the visual input by learning a conditional random field (CRF). Finally, they expressed the generation of natural language as a machine translation problem [8]. Thomason et al. obtained the confidence of the target, action, and scene in the video through the visual recognition system and estimated the most likely subject, verb, object, and place with the factor graph model (FGM) [9]. However, these methods rely too much on predefined templates and detected visual elements and can only simply describe the video, lacking the ability to express semantics.

With the development of the convolutional neural network in the image classification task, three-dimensional convolutional neural network in the video analysis task, and cyclic neural network in the machine translation task, many researchers apply the deep neural network to the video description task. Donahue et al. proposed the long-term recurrent convolutional network (LRCN) model, which can directly generate word sequences through the cyclic neural network without considering the syntax problem of generating description statements [10]. S. Venugopalan et al. proposed a video description model based on LSTM, but this method only considers the characteristics of video frames and ignores the dynamics and continuity of the video [11]. S. Venugopalan et al. proposed a two-stage video description framework, which is composed of a multichannel video encoder and a language decoder that generates sentences. The encoded features are combined by using the fusion layer, and the obtained features are input into the language decoder into a series of words [12]. C. Zhang and Tian et al. proposed a long-term and short-term memory network with visual semantic embedding, which can explore the embedding of learning LSTM and visual semantics [13]. The method proposed by Yao et al. considers the local action features of the video when generating the video description, uses the three-bit convolutional neural network to extract the features of the video clip as the local action features of the video, uses the two-dimensional convolutional neural network to extract the appearance features of the video, and combines the temporal attention (TA) to explore the global time structure of the video [14, 15]. These video description methods only consider visual features and ignore the rich semantic information in the video. Semantic concepts are highly related to the visual content and are widely used in visual recognition tasks.

To sum up, although the research on video description methods has made good achievements, there is still much room for improvement in video feature extraction, video timing features, and video multilingual text description. In view of this, this paper describes the volleyball video by combining the long-term and short-term memory network and attention mechanism. By paying attention to the significant areas in the video, the model can quickly identify the target, effectively solve the lack of visual information, and has a good video description effect when the visual sensor processes the volleyball video.

## 3. Volleyball Video Description Model Based on the Long-Term and Short-Term Memory Network and Attention Mechanism

### 3.1. Long-Term and Short-Term Memory Network

As an improved structure of the ordinary cyclic neural network, long-term and short-term memory network (LSTM) can deal with variable input and output sequences and can effectively avoid the problem of gradient disappearance [16]. The LSTM unit outputs the hidden state *h*_*t*_ of step *t* by relying on the input *x*_*t*_ of the current step *t* and the hidden state *h*_*t*−1_ of time *t* − 1 of the previous step. In the LSTM unit, the flow of input information of the current step and historical memory information is controlled by the input gating and forgetting gating unit. The calculation method is as follows:

(1) $$\begin{array}{c} i_{t}=\sigma \left(W_{xi}x_{t}+W_{hi}h_{t-1}+b_{i}\right),\\ f_{t}=\sigma \left(W_{xf}x_{t}+W_{hf}h_{t-1}+b_{f}\right),\\ o_{t}=\sigma \left(W_{xo}x_{t}+W_{ho}h_{t-1}+b_{o}\right),\\ g_{t}=f\left(W_{xg}x_{t}+W_{hg}h_{t-1}+b_{g}\right),\\ c_{t}=f_{t}⊙c_{t-1}+i_{t}⊙g_{t},\\ h_{t}=o_{t}⊙\Phi \left(c_{t}\right). \end{array}$$

In the formula, *σ* is the sigmoid activation function, Φ is the tanh activation function, ⊙ represents the point multiplication operation of the vector, and the weight matrix *W*_*ij*_ and offset vector *b*_*j*_ are trainable parameters.

Aiming at the problem of automatic generation of video description, based on the LSTM cyclic neural network, by predicting the feature sequence (*x*_1_, *x*_2_,…, *x*_*n*_) of a given input video, the conditional probability of the output word sequence (*y*_1_, *y*_2_,…, *y*_*m*_) is

(2) $$\begin{array}{c} p\left(y_{1},y_{2},\ldots ,y_{m}|x_{1},x_{2},\ldots ,x_{n}\right). \end{array}$$

**Figure 1:**
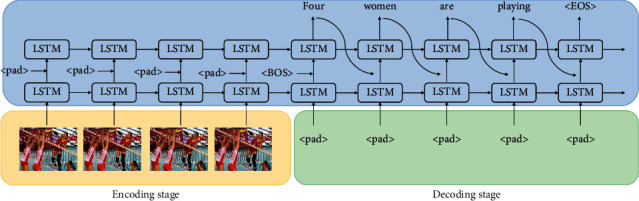
Network video description model based on long-term and short-term memory.

The LSTM model is based on the encoder-decoder framework, and its structure is shown in Figure 1. In the encoding phase, the LSTM layer of the encoder uses the input sequence *X*(*x*_1_, *x*_2_,…, *x*_*n*_) to calculate the intermediate hidden state (*h*_1_, *h*_2_,…, *h*_*n*_) [17]. In the decoding stage, the conditional probability is predicted through the LSTM layer and softmax output layer of the encoder. By linking the probability of each step, the conditional probability of the given input sequence *X* and the output sequence *Y* is obtained as follows:

(3) $$\begin{array}{c} p\left(y_{1},\ldots ,y_{m}|x_{1},\ldots ,x_{n}\right)=\prod_{t=1}^{m}p\left(y_{t}|h_{n+t-1},y_{t-1}\right). \end{array}$$

In the model training stage, the parameters of the model are updated by maximizing the log likelihood probability, i.e.,

(4) $$\begin{array}{c} \theta^{*}=\underset{\theta }{\mathrm{argmax}}\overset{m}{\underset{t=1}{\sum }}\log p\left(y_{t}|h_{n+t-1},y_{t-1};\theta \right). \end{array}$$

*θ* represents the parameters of the model, and the optimization method adopts the random gradient descent method.

### 3.2. Attention Mechanism

**Figure 2:**
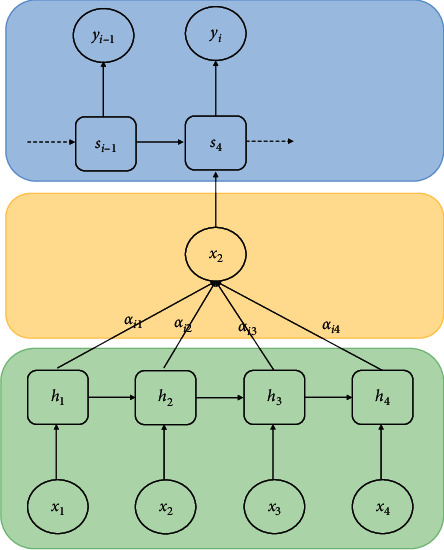
Calculation diagram of the attention mechanism.

The encoder-decoder framework combined with the attention mechanism can learn automatic alignment and translation in the training process of the model. When generating new target short language words, it can find the location of relevant source words, and then the decoder combines the content vector obtained from these locations and the generated target words to predict the target words to be generated [18]. The biggest difference between this method combining the attention mechanism and the basic encoder-decoder method is that it does not need to encode the whole input sentence into a single fixed-length vector, but encodes the input sentence into a vector sequence and dynamically selects a subset of the vector sequence to form a new content vector at each step of the decoding process to generate words at the target end [19]. The calculation method of the dynamic content vector combined with the attention mechanism is shown in Figure 2.

For step *i* of the decoding process, the content vector *c*_*i*_ is weighted by the hidden state sequence (*h*_1_, *h*_2_,…, *h*_*T*_) output by the encoder and the attention weight *a*_*ij*_:

(5) $$\begin{array}{c} c_{i}=\sum_{j=1}^{T}\alpha_{ij}h_{j}. \end{array}$$

The calculation method of attention weight *a*_*ij*_ for hidden state *h*_*j*_ is as follows:

(6) $$\begin{array}{c} \alpha_{ij}=\frac{\exp\left(e_{ij}\right)}{\sum_{k=1}^{T}\exp\left(e_{ik}\right)}. \end{array}$$

*e*_*ij*_ here is calculated by a feedforward neural network model for automatic alignment:

(7) $$\begin{array}{c} e_{ij}=a\left(s_{i-1},h_{j}\right). \end{array}$$

In the formula, **s**_*i*−1_ is the hidden state at the time of decoder *t* − 1, and the parameters of the *a*(*q*, *k*) model and other parameters of the translation model are updated through the training process.

### 3.3. Volleyball Video Description Model Combining the Long-Term and Short-Term Memory Network and Attention Mechanism

**Figure 3:**
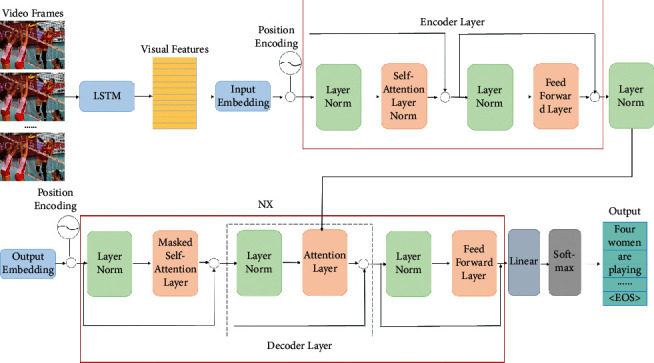
Video intelligent description model combining the long short-term memory network and attention mechanism.

In the task of volleyball video intelligent description, convolutional neural network is usually used to extract image features, and LSTM is used to extract the content vector. The representation ability of the content vector obtained by this method is limited. The attention mechanism can selectively focus on the subset of the video frame sequence to produce the word description of the object or action in the subset of the corresponding frame sequence. Different from the traditional model, the video intelligent description model combined with the long-term and short-term memory network and attention mechanism can dynamically adjust the context vector output by the encoder and realize the function of automatic soft alignment by replacing the convolutional layer and cyclic neural unit layer with the self-attention layer [20]. Its frame is shown in Figure 3.

As can be seen from Figure 3, the video intelligent description model combined with the long-term and short-term memory network and attention mechanism is based on the encoder-decoder framework, which is mainly composed of the encoder, decoder, feature extraction layer at the bottom, embedding layer, linear layer, and softmax layer at the top.

The visual feature extraction layer uses *f*_2 *dC* *NN*_ to represent the visual feature extraction function; then, the sequential multiframe input of a given video is

(8) $$\begin{array}{c} I=\left(I_{1},I_{2},\ldots ,I_{T}\right),\;I_{t}\in {\mathbb{R}}^{h\times w\times c}. \end{array}$$

In the formula, *h*,  *w*,  and *c* are the height, width, and number of channels of the image, and *T* is the sequence length. The visual features are extracted for each frame, respectively:

(9) $$\begin{array}{c} x_{t}=f_{2\;dC\;NN}\left(I_{t}\right). \end{array}$$

The visual feature sequence of consecutive frames can be obtained:

(10) $$\begin{array}{c} X=\left(x_{1},x_{2},\ldots ,x_{T}\right),x_{t}\in R^{d_{\text{feat}}}. \end{array}$$

In the formula, *d*_feat_ is the characteristic dimension. After the visual feature extraction layer, the linear embedding layer is introduced to map the high-dimensional features to the vector of appropriate dimensions for the calculation of the encoder. The calculation method of the embedded layer is

(11) $$\begin{array}{c} x_{t}^{\text{emb}}=W_{\text{img}}x_{t}+b_{\text{img}}, \end{array}$$

and *X*^emb^ ∈ *ℝ*^*T*×*d*_model _^ is obtained, where *d*_model _ is the vector dimension of the query, key, and value in the process of calculating self-attention weight. The calculation method of the frame position information coding layer is as follows:

(12) $$\begin{array}{c} X^{\text{enc}}=X^{\text{emb}}+W_{\text{PE}}. \end{array}$$

Here is the encoded sequence position information, which can be obtained by artificially setting rules and fixed conversion functions. The position information constructor in this paper is

(13) $$\begin{array}{c} W_{\text{PE}}\left(t,2i\right)=\sin\left(t/10000^{2i/d_{\text{modd }}}\right),\\ W_{\text{PE}}\left(t,2i+1\right)=\cos\left(t/10000^{2i/d_{\text{modd }}}\right). \end{array}$$

The trigonometric functions here have different frequencies for features in the same position and different dimensions; for features in different positions of the same dimension, their phases are different. The reason for using the trigonometric function is that the characteristics of the relative position can be described by linear transformation, so it can express the information of the relative position to a certain extent, and trigonometric functions with different frequencies introduce diversified expression of position information.

In the model, the self-attention module adopts the multihead attention mechanism. Compared with the dot-product attention, the feature expression ability of this mechanism is more diverse, and its calculation process is [21]

(14) $$\begin{array}{c} {\text{head}}_{i}=\text{Attention}\left(QW_{i}^{Q},KW_{i}^{K},VW_{i}^{V}\right),\\ \text{MultiHead}\left(Q,K,V\right)={\text{Concat}}_{i=1,\ldots ,h}\left({\text{head}}_{i}\right)W^{O}. \end{array}$$

In the formula, *h* is the number of “heads” in multiple heads, and *W*_*i*_^*Q*^ ∈ *ℝ*^*d*_model _×*d*_*q*_^, *W*_*i*_^*K*^ ∈ *ℝ*^*d*_model _×*d*_*k*_^,  and *W*_*i*_^*V*^ ∈ *ℝ*^*d*_model _×*d*_*v*_^ are trainable parameters. The self-attention module mainly includes normalization, self-attention layer, and residual connection. The forward calculation process of the self-attention module of layer *l* can be expressed as follows:

(15) $$\begin{array}{c} Q^{\left(l\right)}=\text{ LayerNorm }\left(X^{\left(l-1\right)}\right)W_{Q_{e}}^{\left(l\right)},\\ K^{\left(l\right)}=\text{ LayerNorm }\left(X^{\left(l-1\right)}\right)W_{K_{e}}^{\left(l\right)},\\ V^{\left(l\right)}=\text{ LayerNorm }\left(X^{\left(l-1\right)}\right)W_{V_{e}}^{\left(l\right)},\\ f_{\text{self}-\text{att }}\left(X^{\left(l-1\right)}\right)=X^{\left(l-1\right)}+\text{ MultiHead }\left(Q^{\left(l\right)},K^{\left(l\right)},V^{\left(l\right)}\right), \end{array}$$

where *W*_*Q*_*e*__^(*l*)^ ∈ *ℝ*^*d*_model _×*d*_*q*_^, *W*_*K*_*e*__^(*l*)^ ∈ *ℝ*^*d*_model _×*d*_*k*_^,  and  *W*_*V*_*e*__^(*l*)^ ∈ *ℝ*^*d*_model _×*d*_*v*_^ are trainable parameters that transform the output of the previous layer into a triple of query, key, and value. LayerNorm represents the normalization function. Layer normalization normalizes the characteristic scale. Combined with the scaling operation in the point product attention mechanism, the numerical value of the whole calculation process is more stable, and the convergence speed is faster during training.

## 4. Research on the Video Description Effect Combining the Long-Term and Short-Term Memory Network and Attention Mechanism

### 4.1. Datasets and Evaluation Indicators

The experiment selects two commonly used datasets for video description generation to verify the effectiveness of the model; they are MSVD and MSR-VTT datasets.

  Microsoft Research Video Description (MSVD): this dataset contains 1970 video clips. Each video clip describes a single activity, with a duration of 10 s to 25 s and an average length of about 9 s [22]. This paper selects the first 1200 video clips of the dataset as the training set, the next 100 clips as the verification set, and the remaining 670 clips as the test set.
  Microsoft Research Video to Text (MSR-VTT): the dataset contains 10000 video clips and 20 video types [23]. Using the public dataset division method, 6513 video clips are selected as the training set, 497 clips as the verification set, and 2990 clips as the test set.

In order to objectively represent the quality of text description generated by the algorithm, this paper selects four different objective evaluation methods to test the performance of the algorithm, which are BLEU@4, ROUGE-L, METEOR, and CIDEr. To measure the proximity between the generated description text and the manual description text, the ROUGE-L index tends to calculate the recall rate, the METEOR index is applicable to the field of machine translation, and CIDEr is used to evaluate the quality of automatic image description [24–27].

### 4.2. Exploration of Parameter *α* of the Additive Fusion Module

**Figure 4:**
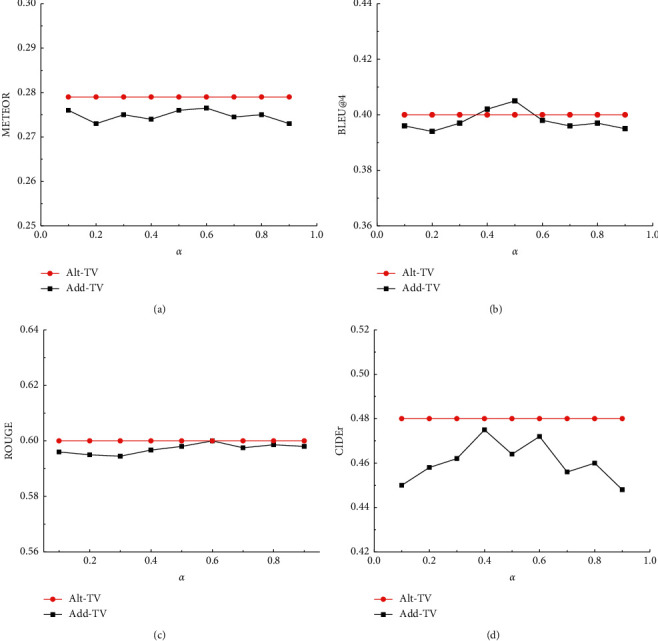
Different parameters in fusion and additive fusion.

In order to verify the effectiveness of the attention fusion module, for the MSR-VTT dataset, *α* with different parameters is selected to compare the performance of the additive fusion module and the attention fusion module. The test results are shown in Figure 4.

Figure 4 shows the comparison between the attention module and the additive fusion module under different parameters. The evaluation results show that when the parameters *α* are adjusted to about 0.4, but its METEOR and CIDEr scores are still lower than those of the attention fusion module. Therefore, compared with the fixed weight ratio, the dynamic attention weight introduced by the attention fusion module is more flexible in fusing multimodal features and can generate higher quality text descriptions.

### 4.3. Comparison with the LSTM Model

**Figure 5:**
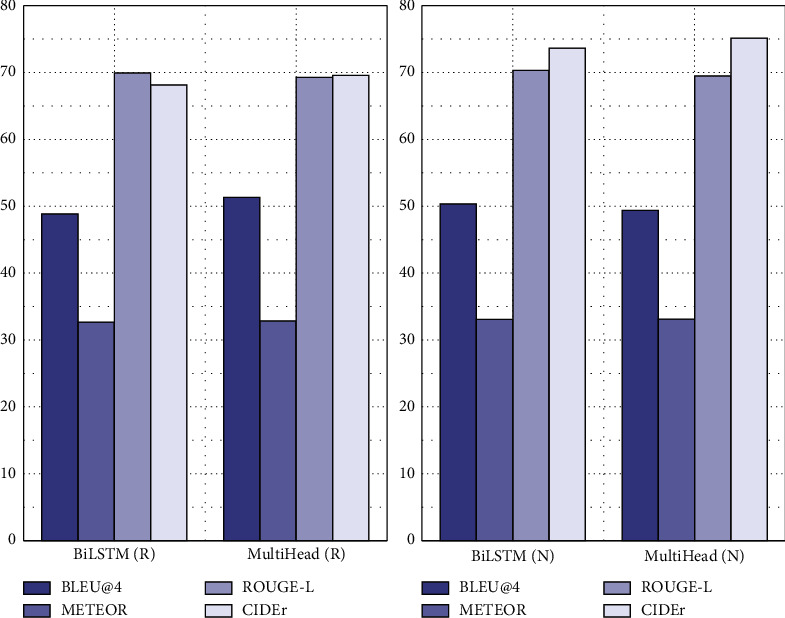
Test results of BiLSTM and multihead models on the MSVD dataset.

**Figure 6:**
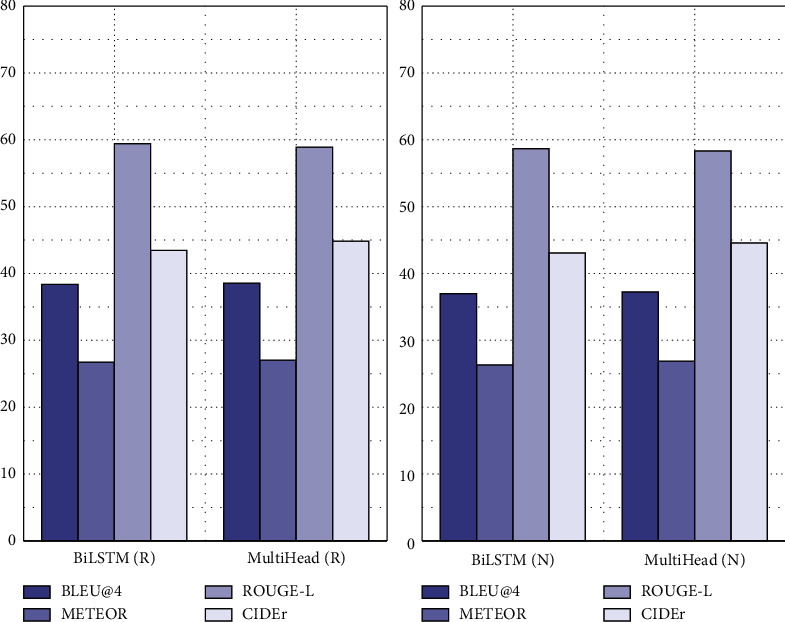
Test results of BiLSTM and multihead models on the MSR-VTT dataset.

In order to verify the performance of the video description model combining the long-term and short-term memory network and attention mechanism, this paper implements a mainstream video description model based on LSTM. Except that the structures of the encoder and decoder are different, the evaluation indexes are compared on MSVD and MSR-VVT datasets when other parameters are set close to the same parameters. In this paper, the model with the attention mechanism is recorded as the multihead model, and the model without the attention mechanism is recorded as BiLSTM. The visual extraction layer is recorded as R using ResNet-152 and N using NASNet. The evaluation results are shown in Figures 5 and 6. The horizontal and vertical dimensions of the graph are the algorithm models used, and the vertical coordinates are the scores of different models.

As can be seen from Figure 5, the METEOR and CIDEr scores of the multihead model on the MSVD dataset are higher than those of the BiLSTM model. These two indicators can also better reflect the quality of text description generation, indicating that the quality of text description generation has been significantly improved after the introduction of the attention mechanism.

As can be seen from Figure 6, in addition to the ROUGE-L score, the other three indicators of the multihead model on the MSR-VTT dataset are higher than those of the BiLSTM model. This is because the introduction of the attention mechanism can make the structure of the visual feature sequence and word sequence more flexible, and better video and sentence content representation can be obtained.

In addition, in the experiment, NASNet, as a visual feature extraction, has greatly improved on the MSVD dataset compared with ResNet-152 and only slightly decreased on the MSR-VTT dataset, which shows that the NASNet pretraining model has a strong generalization ability.

### 4.4. Comparison of Different Parameters of Cluster Search

**Figure 7:**
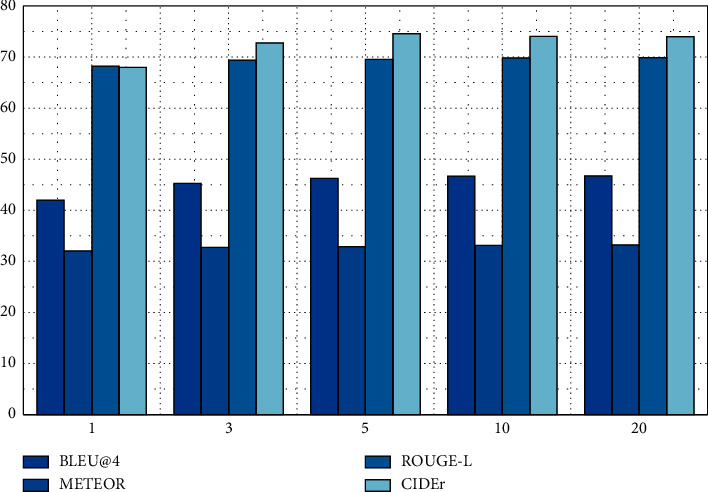
Effects of different beam widths on the quality of text generated by the model when the length penalty coefficient is fixed.

In order to explore the impact of different parameters on the multihead model, this paper explores the impact of different beam widths *k* and length penalty coefficients *α*_*t*_ on the text quality generated by the model on the MSVD test set. First, control the length penalty coefficient *α*_*t*_ = 1.0 to remain unchanged, and change the beam width *k* to 1, 3, 5, 10, and 20, respectively. The evaluation results are shown in Figure 7.

The evaluation results in Figure 7 show the impact of different beam widths on the quality of the generated text. The results show that the generated text can obtain higher evaluation scores with the increase of beam width, but when the beam width increases to more than 5, the gain on scores is relatively small, and the CIDEr score will decrease slightly, which will bring greater search cost. Therefore, a beam width of 5 was used in subsequent experiments.

**Figure 8:**
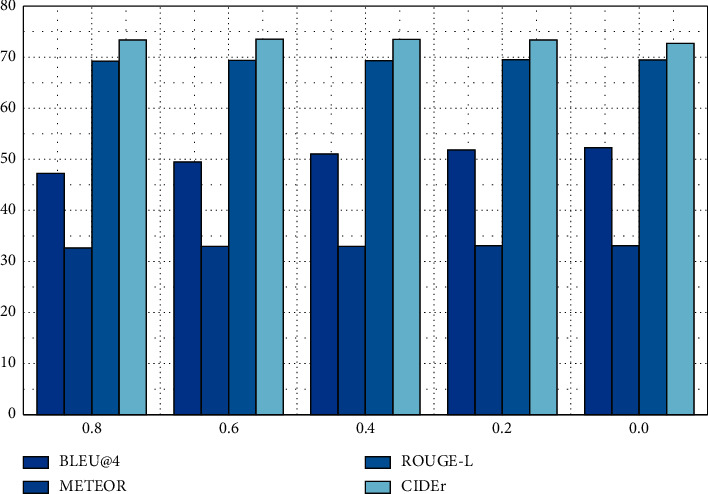
Effects of different length penalty coefficients on the quality of text generated by the model when the beam width is fixed.

The evaluation results in Figure 8 show the impact of different length penalty coefficients on the quality of the generated text. The results show that when the length penalty coefficient is not set or the length penalty coefficient is small, the average sentence length generated is short, which is due to the tendency to output shorter candidate sequences during technical search. BLEU@4 Scores are used to calculate accuracy. The smaller the length penalty coefficient, the higher the score, but this has little effect on other scores.. When the generated sentence is short, the accuracy will be improved because there are fewer 4 tuples in the generated sentence.

### 4.5. Comparison with the Baseline Model

**Figure 9:**
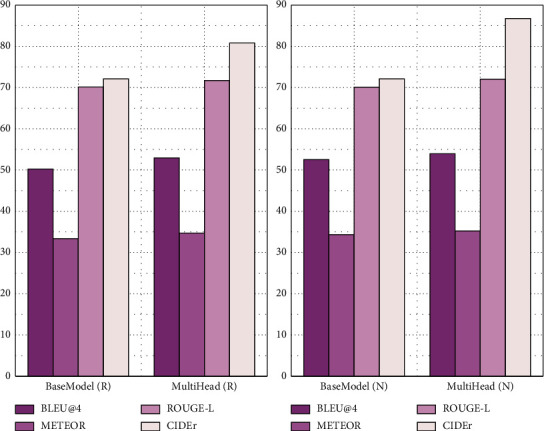
Test results of BaseModel and multihead models on the MSVD dataset.

**Figure 10:**
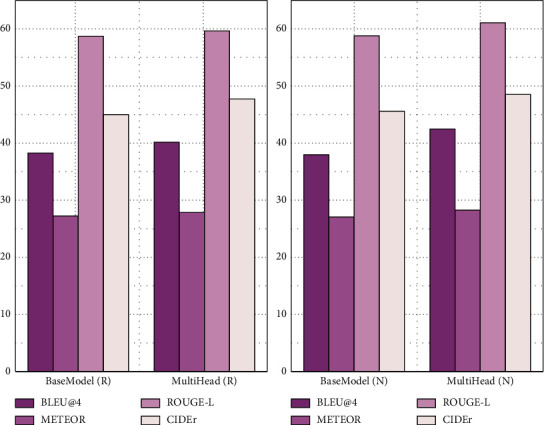
Test results of BaseModel and multihead models on the MSR-VTT dataset.

This section is to verify the effectiveness of the multihead model combined with the long-term and short-term memory network and attention mechanism model and compare it with baseline model BaseModel on MSVD and MSR-VTT datasets, respectively. The test results are shown in Figures 9 and 10, respectively.

As can be seen from Figure 9, the static visual features extracted by NASNet on the MSVD dataset are greatly improved compared with ResNet-152. The index scores of the multihead model are better than those of BaseModel, which shows that the method proposed in this paper has a significant improvement in the quality of text description compared with the baseline model.

As can be seen from Figure 10, NASNet and ResNet-152 have the same performance on the MSR-VTT dataset. The index scores of the multihead model are significantly higher than those of BaseModel, which shows that the method proposed in this paper has certain generalization ability and significantly improves the quality of text description compared with the baseline model.

## 5. Conclusion

In this paper, a video sensor processing method combining the long-term and short-term memory network and attention mechanism is proposed for the intelligent description of the volleyball video. The introduction of the attention mechanism can make the model pay much attention to the important areas in the image/video when generating sentences, quickly identify targets, and effectively solve the problem of visual information loss. Through the comparative experiments of different models, the results show that the dynamic attention weight introduced by the attention fusion module is more flexible than the fixed weight and can generate higher quality text description. Compared with LSTM and base model, the multihead model proposed in this paper combines the long-term and short-term memory network and attention mechanism, scores higher in various evaluation indicators, and significantly improves the quality of the intelligent text description of the volleyball video. The model has a strong generalization ability and good performance in the intelligent description of the volleyball video.

## Data Availability

The data used to support the findings of this study are available from the corresponding author upon request.
